# The Role of RIPK1 and RIPK3 in Cardiovascular Disease

**DOI:** 10.3390/ijms21218174

**Published:** 2020-10-31

**Authors:** Elise DeRoo, Ting Zhou, Bo Liu

**Affiliations:** Department of Surgery, Division of Vascular Surgery, University of Wisconsin School of Medicine and Public Health, Madison, WI 53705, USA; ederoo@wisc.edu (E.D.); zhout@surgery.wisc.edu (T.Z.)

**Keywords:** RIPK1, RIPK3, necroptosis, atherosclerosis, stroke, myocardial infarction, abdominal aortic aneurysm, venous thrombosis

## Abstract

Cardiovascular diseases, including peripheral arterial and venous disease, myocardial infarction, and stroke, are the number one cause of death worldwide annually. In the last 20 years, the role of necroptosis, a newly identified form of regulated necrotic cell death, in cardiovascular disease has come to light. Specifically, the damaging role of two kinase proteins pivotal in the necroptosis pathway, Receptor Interacting Protein Kinase 1 (RIPK1) and Receptor Interacting Protein Kinase 3 (RIPK3), in cardiovascular disease has become a subject of great interest and importance. In this review, we provide an overview of the current evidence supporting a pathologic role of RIPK1 and RIPK3 in cardiovascular disease. Moreover, we highlight the evidence behind the efficacy of targeted RIPK1 and RIPK3 inhibitors in the prevention and treatment of cardiovascular disease.

## 1. Introduction to RIP1 and RIPK3 and Necroptosis

Receptor interacting serine/threonine–protein kinase 1 (RIPK1) and receptor interacting serine/threonine–protein kinase 3 (RIPK3) are intracellular signaling proteins known to play an important role in necroptosis. Necroptosis is defined as a lytic form of cell death, characterized by rapid loss of plasma membrane integrity and release of proinflammatory intracellular contents [1]. It stands in contrast to apoptosis, a caspase-mediated controlled form of cell death that avoids plasma membrane rupture and is immunologically quiet. Necroptosis and necrosis share the common features of plasma membrane permeabilization, release of damage associated molecular patterns (DAMPs), and the provocation of an immune response. However, unlike necrosis, necroptosis is highly regulated by cell signaling pathways [2]. As a result, necroptosis has been called “programmed necrosis”. In experimental settings, necroptosis occurs when pro-apoptotic caspase enzymes are exhausted or inhibited [3]. In a landmark study performed in 1997, Hirsch et al. showed that the caspase inhibitor ZVAD.fmk could not only prevent apoptosis, but also promoted a switch to necrosis [4]. While not characterized in the initial study, the authors suggested that the early stages of apoptosis and necrosis may share a common signaling pathway [4]. This theory has subsequently gone on to be validated. Although necroptosis has been documented in multiple human diseases and their animal models, why death signals trigger necroptosis instead of apoptosis remain unclear. 

**Figure 1:**
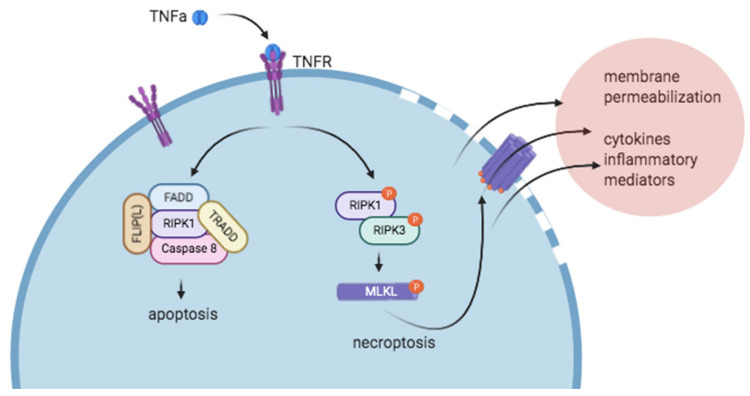
Simplified version of necroptosis signal transduction events downstream of tumor necrosis factor-α/tumor necrosis factor receptor (TNFα/TNFR) interactions. Soluble TNFα binds TNFR and can trigger the formation of a pro-apoptotic (left, complex I) or pro-necroptotic (right, complex IIb) complex. In the absence of caspase-8 and presence of RIPK3, the pro-necroptotic complex IIb forms. After phosphorylation of RIPK1 and RIPK3, RIPK3 phosphorylates MLKL, which subsequently oligomerizes and is thought to insert into the cell membrane, forming pores. After cell membrane permeabilization, ion flux occurs, and intracellular contents are relapsed into the extracellular environment. Figure created with BioRender.com.

Inflammatory mediators such as TNFα, IFNγ, endotoxins, and FasL, signaling through TNFR1, IFNR, TLR3/4, and Fas/TRAILR can all initiate the signaling cascade that leads to necroptosis, although the TNFα/TNFR1-driven signaling cascade has been the most thoroughly investigated [5,6,7,8]. Downstream of TNFα-TNFR1 interactions, three unique complexes (Complex I, Complex IIa, Complex IIb) can form with varying consequences, from cell survival to apoptosis and necroptosis. Complex I, in which ubiquitinated RIPK1 signals through NF-κβ, serves to promote cell survival. Complex IIa, in which de-ubiquitinated RIPK1 interacts with caspase-8, drives apoptosis. Finally, Complex IIb, which forms in the absence of caspase-8, is known as the “necrosome” and promotes necroptosis. Complex IIb consists of activated, phosphorylated RIPK1 and RIPK3, which go on to phosphorylate the pseudokinase Mixed Lineage Kinase Domain like protein (MLKL), a key driver of necroptosis. The interactions between these pathways are complex, and the activation of one pathway can have a regulatory effect on signaling through the other pathway. For example, mice lacking the pro-apoptotic proteins FADD or caspase-8 die of uncontrolled necrosis, which can be rescued by RIPK1 or RIPK3 deletion [8,9,10,11]. After phosphorylation, MLKL oligomerizes and is responsible for permeabilization of the plasma membrane, causing spillage of damage-associated molecular patterns (DAMPs), cytokines, calcium and sodium influx, local inflammation, and cell death [2,5,12] (Figure 1). Preclinical animal models of disease in which cell death is known to play an important role suggest that necroptosis plays a pathologic role in diseases such as atherosclerosis, myocardial infarction, ischemia reperfusion injury, stroke, abdominal aortic aneurysm, and venous thrombosis. Necroptosis is thought to play a role in cardiovascular diseases not only by causing cell death, but also by driving inflammation and inflammasome activation. 

## 2. RIPK1 and RIPK3 in Atherosclerosis

Atherosclerosis, with clinical manifestations including ischemic heart disease, stroke, and peripheral vascular disease, is one of the leading causes of vascular disease world-wide [13]. Ischemic heart disease and stroke are the first and second most common causes of death respectively worldwide, and one out of every four deaths in the United States annually can be attributed to heart disease [14,15]. The pathophysiology underlying atherosclerosis involves the development of fatty streaks upon the vascular wall that evolve into lipid and macrophage rich necrotic plaques with thin fibrous caps at risk for rupture and subsequent vessel occlusion. Low-density-lipoprotein (LDL)-rich macrophage and smooth muscle cell (SMC) death in particular contribute to the development of a necrotic plaque [12]. More simply put, atherosclerosis results from imbalanced vascular and inflammatory cell accumulation and removal in the vessel wall [16]. In vulnerable tissue beds, plaque rupture and vessel occlusion can lead to tissue ischemia and infarction (e.g., myocardial infarction, ischemic stroke, and acute limb ischemia). Given the high morbidity and mortality associated with atherosclerotic disease worldwide, a pressing need exists to define the mechanisms that contribute to atherosclerotic plaque growth and rupture. Beyond examining plaques from humans that have been excised surgically or at autopsy, several hyperlipidemia driven mouse models of atherosclerosis that recapitulate elements of human disease have been developed [17]. Unfortunately, mouse models fail to mimic the spontaneous plaque rupture that occurs in humans, and therefore total plaque size/volume and necrotic core size/volume are often used as markers of disease severity in mice [17,18]. In humans and preclinical models of disease, necroptosis has been shown to be activated in atherosclerotic plaques, and the damaging roles of RIPK1 and RIPK3 in atherosclerosis formation are beginning to come to light. 

Elevated levels of RIPK1, RIPK3, and phosphorylated MLKL, the final executioner of necroptosis, have been detected in unstable atherosclerotic plaques from humans [12,19]. Serum starvation of foam cells, which mimics the insufficient supply of nutrients delivered to cells in atherosclerotic plaques, has been shown to significantly increase RIPK1 and RIPK3 protein levels, and MLKL oligomerization [19]. Moreover, oxidized-LDL, which is present in atherosclerotic plaque [20] and engulfed by local macrophages [21], has been found to induce necroptosis in macrophages in a RIPK3-dependent fashion, and upregulate the expression and translation of RIPK1, RIPK3 and MLKL [12,21]. Lin et al. demonstrated in a mouse model of atherosclerosis that mice lacking RIPK3 developed significantly smaller advanced aortic atherosclerotic lesions. Bone marrow transplantation revealed that loss of RIPK3 from bone marrow derived cells was sufficient to recapitulate the athero-protective phenotype observed in *Ripk3^−/−^* mice, suggesting that hematopoietic cell-specific RIPK3 may play an important role in atherosclerosis progression [22]. Finally, mice deficient in phospholipid transfer protein (PLTP), a protein known to correlate with atherosclerosis severity, showed reduced levels of RIPK3 in atherosclerotic plaques in addition to reduced atherosclerotic lesion burden and intra-lesion cell death. Overexpression with a PLTP-containing adenovirus vector conversely increased RIPK3 protein levels detected in murine atherosclerotic plaques, lesion area and intra-lesion cell death [23]. These findings collectively suggest that necroptosis occurs in human atherosclerotic disease and that RIPK1 and RIPK3 contribute to the atherosclerosis pathophysiology. With clinical translation in mind, several groups have investigated the impact of RIPK inhibitors on atherosclerosis progression and severity. 

While RIPK1 deficient animals die shortly after birth, a consequence thought to be due to altered NF-kβ signaling and altered patterns of cell death, the role of RIPK1 in cardiovascular disease has been able to be explored through the use of targeted inhibitors [24,25]. Using atherosclerosis-prone *Apoe^−/−^* mice that were fed a Western diet to induce lesion formation, Karunakaran et al. showed that 6 weeks of treatment with Necrostatin-1 (Nec-1), an inhibitor of RIPK1, significantly reduced ascending and descending aorta atherosclerosis lesion burden [12]. Phosphorylated MLKL burden within atherosclerotic lesions was reduced by Nec-1 treatment, suggesting that the reduction in lesion size may be due to a lower number of cells undergoing necroptosis. Radiolabeled Nec-1 (^123^I-Nec-1) was found to localize to atherosclerotic plaques in hyperlipidemic mice and positively correlated with lesion area measured by Oil-Red-O staining, showing that this inhibitor of necroptosis is indeed uptaken by atherosclerotic plaques. While the effects of RIPK1 and RIPK3 are pleomorphic and include inducing local inflammation, the authors of this study showed no reduction in inflammatory cytokines in Nec-1 treated mice. Importantly, the authors did show that the athero-protective effects of Nec-1 were independent of lipid burden, as total plasma cholesterol and mouse body weight were not altered by Nec-1 treatment. In the future, studies investigating the impact of Nec-1 treatment on atherosclerotic plaque burden and necrotic core size in *Ripk3^−/−^* (necroptosis null) mice may be of interest to confirm that athero-protective effects of Nec-1 are indeed secondary to decreased necroptosis [12].

An et al. have also investigated the mechanism underlying the athero-protective effects of Nec-1 in vascular cells. In a study performed in human umbilical vein endothelial cells (HUVECs), An et al. found that oxidized-LDL upregulates RIPK1 mRNA and protein, and decreased eNOS production, increased inflammatory vascular adhesion markers, and induced adhesion of monocytes to endothelial cells [21]. The administration of Nec-1 was able to ameliorate the detrimental effects of oxidized-LDL on eNOS expression, decreased vascular adhesion marker expression, and reduced the number of monocytes bound to HUVECs. Importantly, the authors also evaluated the effects of oxidized-LDL administration RIPK1 siRNA treated cells. RIP1-silenced cells showed an attenuated, although not eliminated, increase in vascular adhesion markers and adhesion to monocytes in response to oxidized-LDL treatment [21]. The effects of treatment are intriguing and suggest that RIPK1 may potentially act to promote atherosclerosis through pathways beyond necroptosis.

After identifying that RIPK1 and RIPK3 inhibition can reduce atherosclerotic lesion size and necrotic core and that phosphorylated MLKL is present in human atherosclerotic plaque, Rasheed et al. pursued an investigation into the role of MLKL in atherosclerosis [26]. The authors found that while knockdown of *Mlkl* expression using antisense oligonucleotides in atherosclerosis prone (*Apoe^−/−^*) mice did not decrease total plaque size, *Mlkl* knockdown did decrease necrotic core size and cell death in advanced atherosclerotic lesions. That *Mlkl* knockdown can reduce necrotic core size is an important finding, as necrotic core size is a known marker of plaque vulnerability (i.e., plaques that are at high risk of rupture and thrombosis), and a frequent feature of ruptured plaques [18]. The role of MLKL in atherosclerosis is, however, seemingly complex. Interestingly, *Mlkl* knockdown surprisingly promoted macrophage lipid accumulation and was associated with a weak trend toward increased early atherosclerotic lesion size, although this trend failed to reach statistical significance. Furthermore, knockdown of *Mlkl* was found to have a protective effect on circulating lipid levels, unlike inhibition of RIPK1. The authors argue that the mixed phenotype in the setting of *Mlkl* knockdown is likely secondary to defects in endosomal trafficking [26]. Non-necroptosis roles of MLKL have been previously identified, including inflammasome activation and endosomal trafficking, and likely explain this unexpected finding [27,28,29]. Further investigation into the role of MLKL in atherosclerosis is merited.

## 3. RIPK1 and RIPK3 in Myocardial Infarction

The prevalence of coronary artery disease (CAD) and myocardial infarction (MI) remains a leading cause of morbidity and mortality for patients in the Western hemisphere [14]. Myocardial infarction is driven by an acute or sub-acute vascular occlusion causing tissue hypoxia and subsequent infarction. As such, significant research has been performed investigating the mechanisms that drive cell death and cardiac injury in MI. In the two last decades, the pathologic role of necroptosis in coronary artery disease has come to light, and the contributions of RIPK1 and RIPK3 to acute MI have emerged. 

RIPK1 and RIPK3 mRNA and phosphorylated protein levels have been found to be significantly elevated in cardiac tissue from rodents after myocardial infarction [30,31]. With translation to clinical applications likely in mind, much of the early research into the role of RIPK1 and RIPK3 in MI focused on the efficacy of inhibitors of necroptosis. Inspired by a landmark study published by Degterev et al. showing that Nec-1 could decrease infarct size after middle cerebral artery occlusion [32], Smith et al. investigated the role of RIPK1 in MI and the efficacy of Nec-1 in decreasing MI severity. Nec-1 treatment significantly reduces peroxide-induced cell death in myocytes [33]. Importantly, systemic administration of Nec-1 in rodent models of MI has been shown to significantly reduce necrotic cell death in cardiac tissue without altering apoptotic cell death [34]. Smith et al. also showed that Nec-1 treatment at a dose of 30 μM significantly reduced infarct size in a C57BL/6J mouse model of myocardial infarction (LAD ligation) [33]. The efficacy of Nec-1 in reducing myocardial infarct size in rodent models of MI has subsequently been re-demonstrated by multiple research groups [30,34]. In addition to reductions in necrosis and infarct size, in the presence of Nec-1 treatment reductions in inflammatory cell infiltration are observed in cardiac tissue from rodents after MI [34]. Oerlemans et al. advanced our understanding of the long-term effects of Nec-1 administration in MI by following mice for 28 days after the ischemic insult. At 28 days post MI, Nec-1-treated mice had significantly improved ejection fractions and less adverse cardiac remodeling as determined by MRI [34].

Attention has also been paid to the role of RIPK3, which lies downstream of RIPK1 in the necroptosis signaling cascade, in myocardial infarction. In 2014, Luedde et al. showed in a murine model of myocardial infarction that RIPK3 is significantly upregulated in cardiomyocytes after ischemic insult. Overexpression of RIPK3 in rat cardiomyocytes has been shown to induce necroptosis, as detected by RIP1/RIP3 complex formation and percentage of propidium iodide (PI)-positive cells. Interestingly, RIPK3 overexpression was able to stimulate necroptosis independent of supplemental TNFα, suggesting that necroptosis induction in cardiomyocytes may occur through a pathway independent of TNFα [31]. Notably, signaling pathways that induce necroptosis independent of TNFα have been previously identified [5,35]. Mice deficient in RIPK3 (*Ripk3^−/−^*) had significantly smaller infarct sizes compared to wild-type mice 3 days after MI, and significantly improved ejection fractions (45 ± 3.6 vs. 32 ± 4.4%, *p* < 0.05) and a lesser degree of cardiac hypertrophy compared to control peers 30 days after experimental left anterior descending coronary artery ligation [31,35]. Finally, this decrease in adverse remodeling was associated with decreased inflammation within infarcted cardiac tissue [31]. Interestingly, Zhang et al. found a microRNA termed MiR-325-3p, which is significantly downregulated in mice with MI, that when overexpressed inhibits necroptosis by suppressing RIPK3 expression in myocardial tissue and attenuates the cardiac damage observed after ischemia [36]. Several small molecule compounds have been identified to protect cells against necroptosis through inhibiting RIPK3 kinase activities [37,38,39,40]. Unfortunately, due to cytotoxicity, the efficacy of RIPK3 specific inhibitors in reducing myocardial infarct severity has not been able to be tested.

Several studies suggest that circulating RIPK3 could be a meaningful biomarker in acute myocardial infarction. Kashlov et al. showed that while serum levels of RIPK3 were not significantly elevated at time of presentation in patients suffering from an ST-elevation myocardial infarction (STEMI), serum levels of RIPK3 were significantly elevated along with troponin-I 24 h after onset of STEMI symptoms and percutaneous coronary artery intervention. Interestingly, in patients with normal troponin-I levels prior to PCI, serum levels of RIPK3 and troponin-I were sufficient to differentiate patients with preserved left ventricle ejection fraction (LVEF) compared to those with impaired LVEF. Levels of RIPK3 and troponin-I remained significantly elevated at time of discharge in patients with reduced LVEF (<50%) compared to those with preserved LVEF [41]. In a cross-sectional study of 318 patients with stable coronary artery disease (CAD), unstable angina, and acute myocardial infarction, Hu et al. found that plasma levels of RIPK3 were significantly elevated in patients with CAD (406.87 (311.51, 516.59) pg/mL vs. 241.61 (175.83, 318.13) pg/mL) compared to healthy controls, and that plasma RIPK3 levels positively correlated with CAD severity (acute myocardial infarction > unstable angina > stable CAD). Moreover, they found that plasma levels of RIPK3 are significantly elevated compared to serum levels, perhaps explaining some of the differences in findings compared to the Kashlov et al. study [42]. Being able to gather information about CAD severity in a non-invasive fashion is appealing, as invasive diagnostic procedures such as coronary angiography are not without risks, such as bleeding and vessel injury. Together, these studies suggest that RIPK3 may be able to act as a biomarker for CAD presence and severity.

## 4. RIPK1 and RIPK3 in Stroke

Stroke is one of the leading causes of death and disability annually worldwide [15]. Each year in the United States alone, 795,000 people will suffer from a stroke, and 140,000 people will die as a result of a stroke [43]. While two types of stroke, ischemic and hemorrhagic, account for the majority of stroke cases, ischemic strokes are far more common, and are responsible for 87% of the strokes that occur annually in the United States [43]. Ischemic stroke can further be broken down into atherothrombotic and embolic stroke. An atherothrombotic stroke occurs when a thrombus forms on an atherosclerotic plaque causing tissue ischemia and infarction, while an embolic stroke occurs when a piece of thrombus or plaque dislodges and subsequently blocks blood flow to the brain. While embolic strokes can occur secondary to other sources such as cardiac clot, especially in those with atrial fibrillation, atherothrombotic and atheroembolic ischemic strokes are a major cause of ischemic stroke [44]. The disabilities incurred by stroke are significant, and range from hemiparesis, to hemiplegia, language and speech disorders, impaired vision, and altered levels of consciousness [43]. Given that at its core, tissue ischemia, infarction, and cell death underlie ischemic stroke, the role of necroptosis, RIPK1, and RIPK3 in stroke has become a topic of interest.

Necroptosis has been found to contribute significantly to ischemic stroke both in vitro and in vivo [45]. Multiple independent investigators have found that in vivo and in vitro, ischemia stimulates the upregulation [46] and activation/phosphorylation of RIPK1 [47], RIPK3, and MLKL [47,48,49]. Interestingly, in a study by Gomi Naito et al., ischemia alone was insufficient to upregulate phosphorylated MLKL in the middle cerebral artery occlusion (MCAO) model in mice. Ischemia followed by reperfusion was required for p-MLKL upregulation. This finding merits further investigation, as one of the mainstays of ischemic stroke therapy is tissue plasminogen activator (tPA) administration, which results in reperfusion after a period of ischemia [47]. Using the MCAO model, Gomi Naito et al. were able to show that deficiencies of RIPK1 or RIPK3 reduced ischemic infarct burden. Mice with a kinase dead RIPK1 mutant (*Ripk1^D138N/D138N^*) were found to have reduced stroke volume after 60 min of MCAO followed by 23 h of reperfusion compared to wild-type mice. Intracerebral hemorrhage, which can be seen in humans when ischemic strokes undergo hemorrhagic conversion, was significantly reduced in RIPK1 kinase dead mice and *Ripk3^−/−^* mice. While no difference in early (24 h) stroke volume was seen in *Ripk3^−/−^* mice, stroke volume was significantly lower in RIPK1 kinase dead and *Ripk3^−/−^* mice at 4 days post ischemic insult, and behavior scores were significantly improved compared to wild-type mice [48]. Consistent with the findings of Gomi Naito and colleagues, using a photothrombosis model of cerebral ischemia Yang et al. found that *Ripk3^−/−^* mice had decreased stroke volume at later time points (7 and 14 days after injury) compared to control mice [49]. Interestingly, and unexpectedly, an increase in inflammatory cell infiltration was observed in *Ripk3^−/−^* infarcted tissue. On further exploration, Yang et al. identified that RIPK3 deficiency polarizes macrophages toward the M2, anti-inflammatory phenotype in ischemic cerebral tissues [49].

Studies have found that nec-1 administration protects neurons from ischemic injury [32,45,47,50]. In a study performed by Deng et al., intracerebroventricular administration of Nec-1 30 min before MCAO significantly reduced infarct volume compared to controls. In line with the findings of Gomi Naito et al. in RIPK1 kinase dead mice, Deng et al. found a trend toward reduced neurodeficit scores, and a significant improvement in forelimb placement scores in Nec-1 treated mice [47]. Finally, Deng et al. also showed that Nec-1 treatment significantly reduced IL1β expression in ischemic tissue [47]. While the authors did not investigate whether or not the anti-inflammatory effects of Nec-1 are mediated through inhibition of necroptosis or through an independent pathway, cell death through necrosis is known to stimulate a robust inflammatory response, and it is logical to hypothesize that the anti-inflammatory benefits of Nec-1 are driven by reduced necrotic cell death. Degradation of MLKL after stroke has also been shown to have neuro-protective effects in preclinical models of cerebral ischemia. Zhou and colleagues found, using the murine MCAO model of stroke, that MLKL protein is significantly increased in infarcted tissue 12 to 48 h post ischemia/reperfusion. Moreover, the found that peri-procedure administration of necrosulfonamide (NSA) significantly decreases infarct volume and MLKL protein level, while significantly improving post-stroke neurologic scores [51].

Inhibitors of RIPK3, much like inhibitors of RIPK1 and MLKL, have been shown to reduce ischemic brain injury in pre-clinical models of disease. In an in vitro model of cerebral ischemia, GSK’872, an inhibitor of RIPK3, administered after “ischemia” but during “reperfusion” increased HT-22 cell viability. Similarly, in the MCAO model, infarct volume was significantly reduced by GSK’872 treatment. Interestingly, the authors found that GSK’872 treatment significantly decreased hypoxia-inducible factor-1α (HIF-1α), an important transcriptional factor under hypoxic conditions, and that RIP3 siRNA similarly decreased HIF-1α expression [46]. Dabrafenib, a B-raf inhibitor currently used in cancer therapies that was subsequently found to be a strong inhibitor of RIPK3 at moderate doses [40] has also been found to confer a neuroprotective benefit in cerebral ischemia. Cruz et al. found that prophylactic Dabrafenib administration at a dose of 10 mg/kg before photothrombosis-induced cerebral ischemia significantly reduced infarct size in mice. Importantly, TNFα, an inflammatory cytokine and potentiator of the necroptosis pathway, was significantly reduced in mice with stroke after Dabrafenib treatment [52]. In vitro studies revealed that bone marrow-derived macrophages treated with Dabrafenib produced significantly less TNFα compared to vehicle-treated cells, and thus the authors argue for a model in which Dabrafenib limits stroke injury by reducing cell death, DAMP production, and macrophage/microglia driven TNFα production. That Dabrafenib has already undergone clinical trials in patients suffering from melanoma and is FDA approved for use in cancer therapies makes it an exciting potential therapy in diseases in which necroptosis plays a central role.

## 5. RIPK1 and RIPK3 in Abdominal Aortic Aneurysm

Abdominal aortic aneurysm (AAA) is defined as a greater than 50% dilation in the diameter of the normal aorta [53]. Dilation of the abdominal aorta leads to significant weakening of the arterial wall, and can ultimately lead to rupture [54]. Risk factors for rupture include, but are not limited to, baseline aneurysm diameter and rapid expansion [54]. The annual risk of rupture for patients with aneurysms <5 cm is <1%, but the annual risk of rupture increases substantially for patients with aneurysms >5 cm (5.0–5.9 cm annual risk of rupture 1–11%, 6.0–6.9 cm annual risk of rupture 10–22%, >7.0 cm annual risk of rupture >30%) [54,55]. Rates of growth of >0.5 cm in 6 months or >1 cm in 1 year are considered indications for repair [55].

The pathophysiology of aneurysm formation, expansion, and rupture remains incompletely understood, although inflammation, smooth muscle cell death, and extracellular matrix degradation are known to play a significant role [54,56]. Histologic analysis of AAA tissue shows abundant infiltration of inflammatory cells, including CD4+ T cells, B cells, macrophages, neutrophils, mast cells, and NK cells [56,57]. Reactive oxygen species are also abundant in AAA tissue, and appear to be the product of the invading inflammatory cells [56,57]. Degradation of the extracellular matrix by matrix metalloproteinases (MMPs) and elastase, at least in part produced by invading macrophages, has been shown to be associated with aneurysm formation and progression [56]. Smooth muscle cell apoptosis, driven by a Th2 inflammatory cell phenotype, is associated with aneurysm progression [56,57]. Importantly, the critical contribution of smooth muscle cell death by the alternative cell death pathway, necroptosis, has recently come to light.

In 2015, our lab investigated the hypothesis that RIPK3 mediated necroptosis, not just apoptosis, is involved in aortic aneurysm pathogenesis [58]. Immunostaining of tissue from patients with nonruptured AAA undergoing elective open surgical repair showed elevated RIPK1 and RIPK3 staining and diminished smooth muscle actin staining in the smooth muscle cell layer compared to controls (transplant donors). In a preclinical mouse model of AAA (elastase perfusion model), mice with AAA showed increased mRNA expression of RIPK3 compared to controls. A profound protective effect was observed in *Ripk3^−/−^* treated with aortic elastase perfusion: 0 of 9 *Ripk3^−/−^* mice developed aneurysms (defined as 100% increase in aortic diameter) compared to 8 out of 9 *Ripk3^+/+^* treated mice that did develop aneurysms. Histological analysis revealed decreased elastin fragmentation and less smooth muscle cell loss in *Ripk3^−/−^* mice, in addition to a decrease in necrotic cell burden. As observed in other cardiovascular diseases, a pro-inflammatory role for RIPK3 was demonstrated in AAA. Knockdown of RIPK3 expression in aortic smooth muscle cell culture decreased expression of the inflammatory markers IL6, CCL2, TNFα, and Vcam1 after TNFα induced stimulation [58].

The precise signals in aneurysmal tissues that activate RIPK3 and the subsequent necroptosis remain elusive. However, multiple potential triggers of necroptosis, such as TNF- α, IFNγ, and CD95/FasL are elevated in aortic tissues affected by aneurysm [59,60,61,62]. Furthermore, Luo and colleagues recently reported the presence of cytosolic DNA in aortic tissues from patients with ascending thoracic aortic aneurysm and aortic dissection, a pathologic process more likely to occur in aneurysmal tissue and that increases the risk of aortic tissue undergoing aneurysmal degeneration [63]. Cytosolic DNA is associated with activation of the cytosolic DNA sensing adaptor STING (stimulator of interferon genes), and the activation of STING has been found to cause cell death. Lou et al. found, using the Angiotensin II model of AAA, that mice deficient in STING (*Sting^gt/gt^*) were protected from aortic aneurysm, dissection, and rupture, and that levels of phosphorylated RIPK3 and MLKL in treated aortic tissues were significantly decreased in STING deficient mice. Moreover, cultured SMC deficient in STING and treated with the caspase inhibitor zVAD had lower levels of p-RIPK3 and p-MLKL, and were protected from cell death after exposure to hydrogen peroxide. Finally, STING was found to colocalize with p-RIPK3 and p-MLKL in the SMC layer of diseased aortic tissues. On the basis of these findings, the authors posit that STING may activate RIPK3 and necrosis in AAA and aortic dissection through the STING–TBK1 pathway [62]. Ongoing investigations into the diverse stimuli that may activate RIPK3 signaling in aneurysmal and dissected aortic tissues is merited.

Given the findings detailed above, the impact of inhibitors of necroptosis in AAA became of interest [64]. It should be noted that currently no pharmacologic therapies exist to slow aneurysm progression or prevent rupture. Wang et al. went on to show that in the elastase perfusion mouse model of AAA, the RIPK1 inhibitor Necrostatin-1s (Nec-1s), an optimized form of Nec-1, can slow aneurysm growth after aneurysm formation. Nec-1s treatment from 7 to 14 days after aneurysm induction not only significantly slowed aneurysm growth, but also preserved the histological structure of the aorta (decreased elastin disruption and preserved SMC layer), but also decreased inflammatory cell infiltration into the vessel wall [64]. These findings prompted a search for novel inhibitors of necroptosis that could be tested in the context of abdominal aortic aneurysm. In 2019, Zhou et al. identified a dual RIPK1/RIPK3 inhibitor, GSK259307A (GSK’074) with structural similarity to the established RIPK3 inhibitor GSK’843 [65]. GSK’074 was found to be a potent inhibitor of necroptosis at low concentrations, but lacked the cytotoxicity of GSK’843. In contrast to the Wang et al. study in which Nec-1s was administered 7 days after aneurysm induction in the elastase perfusion model, the authors began their investigation of GSK’074 by testing the efficacy of GSK’074 in inhibiting aneurysm growth at time of aneurysm induction using the CaCl_2_ model. The authors found that aortic aneurysm size was significantly decreased, and aortic architecture was better preserved in GSK’074 treated mice. Aortic aneurysm size and incidence was similarly decreased in GSK’074 treated mice using a second preclinical AAA model (AngII *Apoe^−/−^* model) [65].

While the potential of necroptosis inhibitors to prevent aneurysm formation is certainly of interest, finding a drug that can prevent or reverse aneurysm growth after aneurysm diagnosis is of far greater clinical relevance. Khoury et al. therefore sought to investigate the impact of GSK’074 treatment on aneurysm size after aneurysm formation. Using the CaCl_2_ model of AAA, Khoury et al. showed that treatment with GSK’074 from 7 to 28 days after aneurysm induction reduced aneurysm growth and inflammatory cell infiltrate while preserving native aortic elastin and smooth muscle cell structure [66]. Collectively, the findings of Wang, Zhou, and Khoury et al. demonstrate that the necroptosis inhibitors Nec-1s and GSK’074 show clinical promise and should continue to be investigated in the context of AAA.

## 6. RIPK1 and RIPK3 in Thrombosis

Thrombosis, or the activation of the coagulation cascade and formation of a blood clot within the circulatory system, is a nearly ubiquitous feature of the disease processes previously discussed. Thrombosis and thromboembolism play a critical role in myocardial infarction and stroke, and aortic thrombus is invariably present within dilated, aneurysmal aortas in humans. While aortic occlusion from thrombus accumulation in AAA is rare, non-occlusive thrombus within aortic aneurysms is suspected to play a pathologic role in AAA progression, contributing to local vessel wall hypoxia and damage [67,68,69,70,71,72,73]. Thrombosis can also occur in the deep venous system, referred to as a deep vein thrombosis (DVT). Venous thromboembolism (VTE), which encompasses both DVT and pulmonary embolism (PE), is a relatively common disease, with an estimated incidence of 300,000–600,000 events in the United States annually [15,74]. VTE recurs in approximately 30% of patients within 10 years after an event and is associated with not only reduced survival, but significant morbidity and substantial healthcare costs. An estimated $7–10 billion spent annually in the treatment of acute VTE and its complications [15,74]. In the last decade, several studies have identified a link between RIPK1, RIPK3, necroptosis, and thrombogenesis in both the arterial and venous setting.

Early in thrombogenesis, two phases of hemostasis occur: primary hemostasis and secondary hemostasis. Primary hemostasis refers to the activation and aggregation of platelets, with subsequent platelet plug formation. Secondary hemostasis occurs alongside primary hemostasis, and involves activation of the coagulation cascade with subsequent generation of insoluble fibrin [75]. In 2017, Zhang et al. demonstrated that RIPK3 plays a critical role in primary hemostasis by regulating platelet function [76]. RIPK3 was found to be present in platelets from both mice and humans. Murine tail bleeding time, an indicator of primary hemostasis [77,78], was significantly prolonged in *Ripk3^−/−^* mice compared to wild-type littermates. Furthermore, *Ripk3^−/−^* mice had significantly prolonged time-to-occlusion in a ferric-chloride mesenteric arteriole injury model, as monitored by intra-vial microscopy with fluorescence-tagged platelets. Finally, deletion of RIPK3 within platelets caused significant aggregation defects and disrupted dense granule secretion [76]. The findings of Zhang et al. suggest that further investigation into the role of RIPK3 in primary hemostasis and the potential of RIPK3-targeted therapies in thrombotic disease is merited.

Thus far, investigations into the role of RIPK3 in venous thrombosis have been limited. Those that have been performed, however, suggest an important role of the RIPK1/RIPK3/MLKL driven necroptosis pathway in venous thrombosis. Venous thrombogenesis is complex, and involves the cooperation of platelets, monocytes, and neutrophils [79]. NETosis, or the release of an extracellular DNA “net” from neutrophils has been found to be critical to thrombogenesis in pre-clinical models of disease, and relevant in human venous thrombosis [80,81,82,83]. NETosis does not always obligate cell death, as in a process known as “vital-NETosis” some neutrophils have been observed to survive and migrate after release of DNA [84]. The relationship between NETosis and necroptosis remains largely obscure, although currently they are thought of as unique processes.

Given the importance of NETosis in venous thrombosis, and the finding that RIPK3 can contribute to NETosis in crystallopathies such as gout [85], Nakazawa et al. sought to investigate whether or not MLKL, the executor of necroptosis, drives NETosis in the context of venous thrombosis [86]. The authors found, using the murine IVC ligation model of DVT, that MLKL staining in venous thrombi co-localized with markers of NETosis, such as citrullinated histone H3. Importantly, the authors showed that genetic deficiency of MLKL in mice (*Mlkl^−/−^*) or Nec-1s treatment reduced thrombus incidence, size, and intra-thrombus markers of NETosis. Given that activated platelets are drivers of NETosis and the previous findings of Zhang et al. that RIPK3 is critical to several platelet activation pathways, the authors investigated the relationship between RIPK1, MLKL, and platelet activation. Somewhat surprisingly in the context of the findings of Zhang et al., the authors found that Nec-1s and necrosulfonamide, an MKLK inhibitor, treatment of platelets did not influence platelet activation. They did, however, find that Nec-1s and necrosulfonamide decreased TNFα-driven platelet-neutrophil aggregate formation [86]. Given the ready availability of inhibitors of RIPK1/RIPK3 and the large clinical impact of venous thrombosis annually worldwide, the mechanistic relationship between RIPK1/RIPK3/MLKL, platelets, NETosis and venous thrombosis certainly merits further investigation.

## 7. Conclusions

RIPK1 and RIPK3 are kinases that play an essential role in the newly identified cell death pathway of necroptosis and in inflammation. The pathologic role of RIPK1 and RIPK3 in cardiovascular diseases such as atherosclerosis, myocardial infarction, stroke, abdominal aortic aneurysm, and venous thrombosis has come to light over the last two decades, and are summarized in Table 1. With cardiovascular diseases being the number one cause of death globally, taking 17.9 million lives annually, every effort must be made to better understand the cellular and molecular mechanisms that drive cardiovascular disease so that targeted treatments can be developed. Fortunately, inhibitors of receptor interacting protein kinases have already been developed, with some already in clinical use for alternate diseases (Table 2). Research into the role of RIPK1 and RIPK3 in cardiovascular disease and the potential to translate inhibitor studies from the bench to the bedside must continue to be aggressively pursued.

## Figures and Tables

**Table 1:** Summary of Studies Investigating Receptor Interacting Protein Kinases in Cardiovascular Disease.

Disease	Model/Subjects	Inhibitor	Pertinent Findings	Ref.
Atherosclerosis	Human plaques, *Apoe^−/−^* mice, oxLDL BMDM treatment	Nec-1	RIPK3 and MLKL expression & activation increased in human plaques, Nec-1 reduces plaque size/necrotic core in mice, reduced ox-LDL induced necroptosis in BMDM	[12]
Atherosclerosis	Human plaques, in vitro serum starvation foam cell model	None	RIPK1/3 expression increased in plaques, serum starvation increases RIPK1/RIPK3 expression, MLKL oligomerization	[19]
Atherosclerosis	ox-LDL HUVEC treatment	Nec-1	Ox-LDL increases RIPK1 expression & inflammation, nec-1 ameliorates this effect	[21]
Atherosclerosis	*Ripk3^−/−^*; *Ldlr^−/−^* mice, *Apoe^−/−^* mice	None	*Ripk3^−/^Ldlr^−/−^* mice*^−^*mice have significantly smaller advanced plaques	[22]
Atherosclerosis	*Apoe^−/−^* mice	Anti-sense MLKL oligonucleotides	MLKL knockdown decreased necrotic core size but not plaque size, decreased lipid levels	[26]
MI	Rat LAD ligation	Nec-1	RIPK1/RIPK3 increased in cardiac tissue after MI, Nec-1 decreased infarct size	[30]
MI	Mouse LAD ligation, *Ripk3^−/−^* mice	None	RIPK3 increased in cardiac tissue after MI, EF preserved in *Ripk3^−/−^* mice after LAD ligation	[31]
MI	Mouse and rat-derived cardiomyocytes, mouse LAD ligation	Nec-1	Nec-1 reduced peroxide induced cell death, murine infarct size	[33]
MI	Mouse LAD ligation	Nec-1	Nec-1 reduced infarct size, necrotic cell death, prevented adverse remodeling at 28 days	[34]
MI	*Ripk3^−/−^* mice, Mouse LAD ligation	None	Reduced infarct size in *Ripk3^−/−^* mice	[35]
MI	Human STEMI patients	None	In patients with normal troponin on presentation, serum RIPK3 predicts impaired LV function	[41]
MI	Humans with CAD, angina, unstable angina	None	Plasma RIPK3 correlates with CAD severity	[42]
Stroke	Mouse MCAO model	Nec-1	Intracerebroventricular Nec-1 reduced infarct volume	[32]
Stroke	Oxygen-deprived glucose (ODG) in vitro model, MCAO mouse model	GSK’872	ODG and MCAO upregulate RIPK1, RIPK3, MLKL, GSK’872 reduces infarct volume	[46]
Stroke	Rat MCAO model	Nec-1	Ischemia activates RIPK1/3/MLKL signaling. Nec-1 reduces infarct volume	[47]
Stroke	Mouse MCAO model, *Ripk3^−/−^* mice*, Ripk1^D138N/D138N^* mice	None	Inactivation of RIPK1 and absence of RIPK3 can ultimately decrease stroke volume, improve behavioral scores	[48]
Stroke	Mouse MCAO model, ODG in vitro model, *Ripk3^−/−^* and *Mlkl^−/−^* mice	None	RIPK3 or MLKL deficiency decreases stroke size, neurologic deficits, polarizes macrophages to M2 phenotype	[49]
Stroke	Mouse MCAO model, ODG in vitro model	Nec-1	Nec-1 protects cells from ODG related death, Nec-1 reduced infarct volume	[50]
Stroke	Mouse MCAO model	NSA	Decreased infarct size, neurologic deficits, MLKL levels; increased MLKL degradation after NSA treatment	[51]
Stroke	Photothrombosis induced ischemic injury in mouse	Dabrafenib	Dabrafenib reduced infarct size, inflammation	[52]
AAA	Murine elastase perfusion model, *Ripk3^−/−^* mice	None	RIPK1/RIPK3 are locally upregulated in AAA, *Ripk3^−/−^* mice are protected from AAA	[58]
AAA	Murine elastase perfusion model	Nec-1s	Nec-1s slows aneurysm growth, decreases inflammation, preserves vessel architecture	[64]
AAA	Murine CaCl_2_ model, murine AngII *Apoe^−/−^* model	GSK’074	GSK’074 can prevent aneurysm growth, preserve vessel architecture in both aneurysm models	[65]
AAA	Murine CaCl_2_ model	GSK’074	GSK’074 slows aneurysm growth, preserves vessel architecture	[66]
AAA	Murine AngII and CaCl_2_ model, cell culture	None	STING deficiency decreases necroptosis and protects mice from AAA	[62]
Arterial thrombosis	Murine FeCl_3_ injury model, tail bleeding, platelet activity assays, *Ripk3^−/−^* mice	None	*Ripk3*^−/−^ mice have prolonged tail bleeding, FeCl_3_ arteriole time to occlusion, abnormal dense granule secretion	[76]
Venous Thrombosis	IVC ligation model, *Mlkl^−/−^* mice	Nec-1s, NSA	Nec-1s treatment and MLKL deficiency decrease thrombus size, decrease NETosis. Nec-1s and necrosulfonamide decrease platelet-neutrophil aggregation	[86]

AAA, abdominal aortic aneurysm; AngII, angiotensin II; BMDM, bone marrow-derived macrophage; EF, ejection fraction; IVC, inferior vena cava; LAD, left anterior descending artery; LV, left ventricle; MCAO, middle cerebral artery occlusion; MI, myocardial infarction; MLKL, mixed-lineage kinase domain like protein; Nec-1, Necrostatin-1; Nec-1s, Necrostatin-1s; NSA, Necrosulfonamide; oxLDL, oxidized low density lipoprotein; RIPK, receptor interacting protein kinase; STEMI, ST-elevation myocardial infarction.

**Table 2:** ^a^ Summary of Receptor Interacting Protein Kinase Inhibitors Tested in and Beyond Cardiovascular Disease.

Inhibitor Name	Molecular Target	Tested Applications	Use in Clinical Trials: Yes/No	Ref.
Necrostatin-1	RIPK1	Atherosclerosis ^§^, stroke ^§^, MI ^§^	No	[12,21,30,32,33,34,47,50]
Necrostatin-1s	RIPK1	AAA ^§^, venous thrombosis ^§^	No	[64,86]
PN10	RIPK1	TNFα induced SIRS ^§^	No	[87]
cdp27	RIPK1	TNFα induced SIRS ^§^	No	[88]
GSK′963	RIPK1	TNFα induced SIRS ^§^	No	[89]
RIPA-56	RIPK1	TNFα induced SIRS ^§^	No	[90]
GSK2656157	RIPK1	TNFα induced SIRS ^§^	No	[91]
Sibiriline	RIPK1	concanavalin A-induced hepatitis ^§^	No	[92]
GSK’872	RIPK3	Stroke ^§^,	No	[46]
GSK’074	RIPK1 & RIPK3	AAA ^§^,	No	[65,66]
DNL747	RIPK1	Alzheimer’s disease, ALS, MS	Yes- Phase I	[87]
GSK2982772	RIPK1	Psoriasis, UC, RA	Yes- Phase II	[93]
Dabrafenib	RIPK3	Stroke ^§^, Metastatic melanoma	Yes- Metastatic melanoma, FDA approved	[52,87]
Ponatinib	RIPK1&RIPK3	TNFα induced SIRS ^§^	Yes- FDA approved for CML and Ph+ALL	[87,94]
Sorafenib	RIPK1&RIPK3	TNFα induced SIRS ^§^ and renal ischemia–reperfusion injury ^§^	Yes- FDA approved for advanced liver cancer; renal cancer; thyroid cancer	[95]

^§^ Tested in laboratory context, not in humans. ^a^ Clinical trial data gathered from clinicaltrials.gov. AAA, abdominal aortic aneurysm; ALS, amyotrophic lateral sclerosis; MI, myocardial infarction; MS, multiple sclerosis; RA, rheumatoid arthritis; RIPK, receptor interacting protein kinase; UC, ulcerative colitis; SIRS, systemic inflammatory response syndrome; CML, chronic myeloid leukemia; Ph+ ALL, Philadelphia chromosome positive acute lymphoblastic leukemia.

## Abbreviations

AAA	abdominal aortic aneruysm
ALS	amyotrophic lateral sclerosis
AngII	angiotensin II
APOE	apolioprotein E
CAD	coronary artery disease
CML	chronic myeloid leukemia
DAMP	damage-associated molecular patterns
DVT	deep vein thrombosis
FADD	FAS-associated death domain
HIF1α	hypoxia inducible factor-1α
HUVEC	human umbilical vein endothelial cells
IFNγ	interferon γ
IL1β	Interleukin 1β
IVC	inferior vena cava
LAD	left anterior descending
LDL	low-density lipoprotein
LVEF	left ventricle ejection fraction
MCAO	middle cerebral artery occlusion
MI	myocardial infarction
MLKL	mixed-lineage kinase domain like protein
MMP	matrix metalloproteinase
MS	multiple sclerosis
Nec-1/1s	necrostain-1/1s
NET	neutrophil extracellular trap
NFKβ	Nuclear factor kappa β
NSA	necrosulfonamide
PCI	percutaneous coronary intervention
PE	pulmonary embolism
Ph+ ALL	Philadelphia chromosome positive acute lymphoblastic leukemia
PI	propidium iodide
PTLP	phospholipid transfer protein
RA	rheumatoid arthritis
RIPK1	receptor interacting protein kinase 1
RIPK3	receptor interacting protein kinase 3
SIRS	systemic inflammatory response syndrome
SMC	smooth muscle cell
STEMI	ST elevation myocardial infarction
STING	stimulator of interferon genes
TLR 3/4	toll like receptor 3/4
TNFα	tumor necrosis factor α
TNFR1	tumor necrosis factor receptor 1
TRAILR	tumor necrosis α related apoptosis inducing ligand receptor
tPA	tissue plasminogen activator
UC	ulcerative colitis
VTE	venous thromboembolism
